# Synthesis of polypyrrole/nitrogen-doped porous carbon matrix composite as the electrode material for supercapacitors

**DOI:** 10.1038/s41598-020-72392-x

**Published:** 2020-09-21

**Authors:** Minzhen Feng, Wei Lu, Yun Zhou, Ranran Zhen, Hongmei He, Ya Wang, Chunmei Li

**Affiliations:** 1 Chongqing Key Laboratory of Inorganic Function Materials, College of Chemistry, Chongqing Normal University, Chongqing, 401331 People’s Republic of China; 2 College of Chemistry and Chemical Engineering, Chongqing University, Chongqing, 400044 People’s Republic of China

**Keywords:** Chemistry, Electrochemistry, Materials chemistry

## Abstract

Polypyrrole complex nitrogen-doped porous carbon matrix (PPy/N-PCM) was synthesized by a simple two-step method. Firstly, graphene oxide was prepared by the modified Hummers method. Secondly, Polypyrrole was compounded on the graphene oxide substrate, and the carbon matrix with a high specific surface area was obtained through high-temperature carbonization and KOH activation, and polypyrrole was used as a nitrogen source for the final nitrogen-doped composite material. The structure characterization of the carbon matrix and the final composite material shows that the carbon matrix surface has obvious porous structure, and the polypyrrole nanospheres grow uniformly on the porous carbon matrix surface. The electrochemical evaluation show that the prepared PPy/N-PCM has excellent supercapacitor performance, and its specific capacitance can reach 237.5 F g^−1^. When the current density reaches 10 A g^−1^, it has good cycle stability (the capacitance retention after 1000 charge and discharge is 88.53% of the initial capacitance value, which is better than pure PPy-60.76% and PPy/rGO-C-71.84%). The excellent capacitance performance, good-looking micro-morphology and simple synthesis method of the PPy/N-PCM provide the possibility for its commercialization.

## Introduction

Supercapacitor is a new type of energy storage device which is environmentally friendly, high power fast charging and high safety. It is above advantages that have attracted the attention which comes from scientific research and industry^1–3^. According to different energy storage mechanisms, supercapacitors can be divided into two categories^4–6^: The first type is an electric double layer capacitor (EDLC) in which the charge storage process doesn’t induce electric current. The electrons don’t migrate on the electrode contact surface, such as carbon nanotubes and graphene^7^. The second type is Faraday quasi-capacitor, also known as the tantalum capacitor. It is a complementary form of the electric double layer capacitor. This tantalum capacitor is generated from some electro-adsorption processes and highly reversible redox reactions of oxides, such as transition-metal oxides or conductive polymers^8^. So for supercapacitors, electrode material play a key factor in determining their performance.

Many different materials are used as the electrode material for supercapacitors, such as polypyrrole, graphene, polyaniline, polyacetylene. Among such materials, polypyrrole (PPy) has the advantages of lower raw material cost than most carbon materials and conductive polymers, simple synthesis and high theoretical tantalum capacitance^9–12^. However, due to the morphology collapse and reduced conductivity of polypyrrole during high rate cycling, it exhibits poor cycle stability and rapidly attenuating capacitance. Therefore, the polypyrrole composite materials have been extensively studied, including polypyrrole/carbon, polypyrrole/metal oxide^13,14^. Among them, polypyrrole/graphene composite has attract much attention^15^. For example, Feng et al.^16^ reported that graphene oxide was modified (MGO) and then compounded with PPy to obtain PPy/MGO composites. At a current density of 1 A g^−1^, the specific capacitance of PPy/MGO in the three-electrode system was 202 F g^−1^, and the energy density was 8.49 Wh kg^−1^. After 1000 cycles, the capacitance retention rate of PPy/MGO was 83.8%. Chang et al.^17^ used an electrochemical synthesis method to prepare graphene oxide/PPy composite membranes. The specific capacitance at a current density of 1 A g^−1^ was 289 F g^−1^, but the cycle stability was not studied. However, the strong Vander Waals force between the graphene sheets tend to cause sheet stacking, which reduces the specific surface area of the graphene and can’t support PPy well^18^. This will reduce the specific capacitance and cycle stability of the polypyrrole/graphene composite. In order to effectively improve the electrochemical performance of electrode materials, three-dimensional porous nitrogen-doped graphene-based composite materials are widely used in the study of supercapacitor electrode materials^19,20^. On the one hand, the three-dimensional porous nitrogen-doped structure can provide a larger specific surface area for the carbon substrate, promoting the diffusion and transfer of the electrolyte ions and electrons^21,22^. On the other hand, the nitrogen is doped into the carbon matrix can enhance the surface activity of the carbon matrix and produce a pseudocapacitive effect^23,24^.

In our work, we added polypyrrole to introduce nitrogen into the graphene oxide matrix, and continued to compound polypyrrole on the nitrogen-doped carbon matrix to avoid the collapse of polypyrrole morphology^25^. Firstly, polypyrrole was self-assembled on graphene oxide template to obtain the polypyrrole/graphene oxide composite material. Secondly, the polypyrrole/graphene oxide composite was then carbonized and activated to obtain the nitrogen-doped porous carbon matrix. In this process, the introduction of nitrogen atoms was from the polypyrrole, which not only solved the problem of graphene matrix aggregation, but also provided an active site for the attachment and deposition of polypyrrole. The porous structure could also increase the specific surface area. Finally, the polypyrrole nanospheres were anchored on the surface of the nitrogen-doped porous carbon matrix. With the aid of the porous structure of the carbon matrix, the morphology stability of polypyrrole was improved. Such polypyrrole/three-dimensional porous carbon matrix has high specific capacitance, good cycle stability, and good morphology, which makes it promising as one of the most popular electrode materials for supercapacitors in the future.

## Experimental section

### Material

Graphite powder (reagent grade), Pyrrole (reagent grade), Polytetrafluoroethylene (reagent grade) were purchased from Adamas-beta (shanghai, China). Ammonium persulfate (APS) and N-Methylpyrrolidone were purchased from Chengdu Kelong Chemical Reagent plant (Chengdu, China). Other chemical reagents used in this work were analytical reagents.

### Preparation of PPy/GO nanocomposite

The graphene oxide (GO) was synthesized by improved Hummers method^26^. After the synthesis, a certain amount of graphene oxide was taken and dispersed in 60 ml of 1 mol L^−1^ HCl solution and 20 ml of ethanol solution. Pyrrole monomer was dripped in the solution and stirred for 30 min. At the same time, ammonium persulfate was dissolved in 20 ml of 1 mol L^−1^ HCl solution. Then the prepared two solutions were slowly mixed and stirred in an ice-water bath overnight. (The molar ratio of pyrrole monomer to APS is 1–1.5). Finally, the resulting solution was suction filtered. The filter cake was washed with HCl, distilled water and ethanol, and then dried at 50 °C overnight to obtain the product^27^.

### Preparation of N-PCM

The PPy/GO composite prepared in the previous step was carbonized at 400 °C for 1 h under the condition of nitrogen protection in tubular furnace at a heating rate of 5 °C min^−1^. After carbonization, the product was taken out and soaked in 1 mol L^−1^ potassium hydroxide solution and stirred, and then fully dried at 70 °C. The dried sample was placed in porcelain boat and further carbonized by gradient heating in a tube furnace (heating rate 5 °C min^−1^, 450 °C 1 h, 600 °C 1 h, 700 °C 2 h). The product was washed with hydrochloric acid to remove impurities and then washed with deionized water until about neutral. The obtained product is an N-PCM (N-doped Porous Carbon Matrix). Since the amount of KOH will affect the pore size and the specific surface area of the carbon matrix, we prepared carbon matrix with different amounts of potassium hydroxide, wherein the mass ratio of KOH to carbon was 0.5:1, 1:1, 2:1, 3:1, respectively, and these products were named as N-PCM-0.5, N-PCM-1, N-PCM-2, and N-PCM-3. For comparison, rGO-C (the carbon matrix obtained by carbonizing PPy/GO composite material without potassium hydroxide treatment) was also prepared^27^.

### Preparation of PPy/N-PCM and PPy/rGO-C composites

**Scheme 1:**
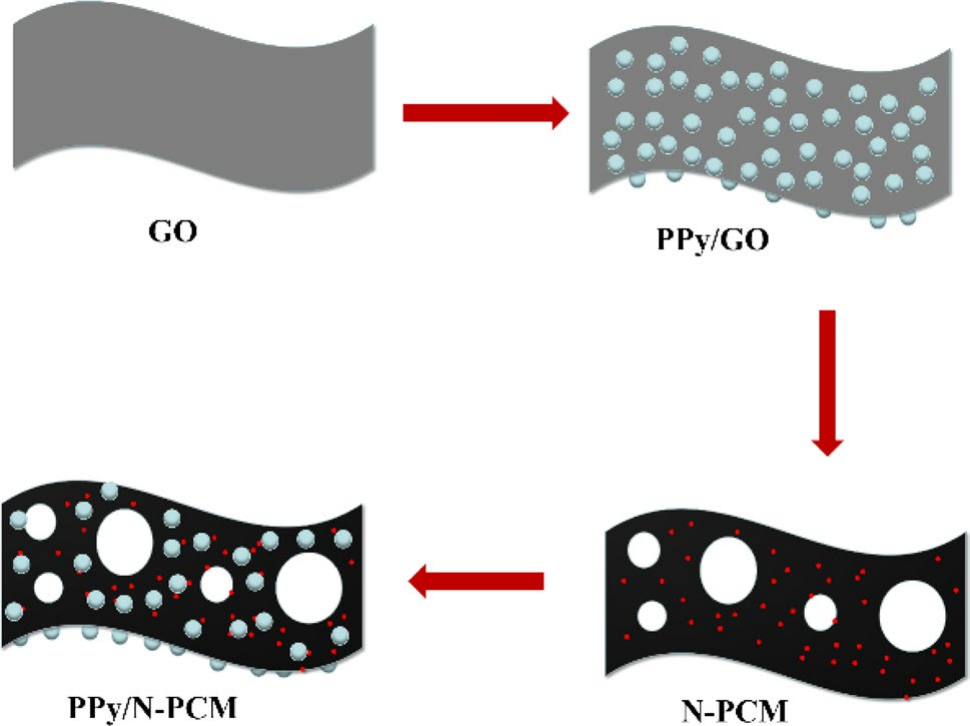
The illustration of the synthetic procedures of PPy/N-PCM.

The PPy/N-PCM composite image is shown as Scheme 1. A certain amount of N-PCM was taken and dispersed in 60 ml of 1 mol L^−1^ HCl solution and 20 ml of ethanol solution. Pyrrole monomer was dripped in the solution and stirred for 30 min. At the same time, ammonium persulfate was dissolved in 20 ml of 1 mol L^−1^ HCl solution. Then the prepared two solutions were slowly mixed and stirred in an ice-water bath overnight. Finally, the resulting solution was suction filtered. The filter cake was washed with HCl, distilled water and ethanol, and then dried at 50 °C overnight to obtain the product^27^. The effect of pyrrole concentration on PPy/N-PCM composite is critical. In this paper, four samples with pyrrole concentrations of 0.02 M, 0.04 M, 0.06 M, and 0.08 M were prepared and recorded as PPy-2/N-PCM, PPy-4/N-PCM, PPy-6/N-PCM, and PPy-8/N-PCM. The preparation process of PPy/rGO-C composite was the same with that of PPy/N-PCM, but the concentration was not discussed.

### Material characterization

The morphology of the samples was characterized by field emission scanning electron microscope (FE-SEM, Tescan Mira 3) with acceleration voltage of 10.0 kV and transmission electron microscope (TEM, Thermo Fisher Scientific Talos F200S) with acceleration voltage of 200 kV. In order to study the composition and structure of the samples. FT-IR characterization was performed on Fourier Transform Infrared Spectroscopy (FT-IR)-8400S at a wavenumber ranging from 500 to 4000 cm^−1^. The Raman test is performed under the conditions of a wavelength of 514 nm and a sampling step length of 1.45 cm^−1^. X-ray photoelectron spectroscopy (XPS) uses an Al (Ka) ray (hv = 1486.6 eV) excitation source, a working voltage of 15 kV, and signal accumulation for 5–10 cycles. The structure of the samples was analyzed, using Cu (Ka) ray and Shimadzu RigakuD/MAX 2550 X-ray diffractometer at a scan rate of 2° min^−1^ and an angle of 2θ = 5°–80°. The specific surface area and pore structure of the samples were measured under 77 K and nitrogen protection by surface area and porosity analyzer (3H-2000PS1).

### Electrochemical test

The electrochemical properties of the prepared samples were investigated at room temperature with three electrode system by using 1 mol L^−1^ H_2_SO_4_ solution as electrolyte. A mixture of electrode material, acetylene black and polytetrafluoroethylene in a mass ratio of 8:1:1 was uniformly coated with N-Methylpyrrolidone on conductive carbon paper as test sample. The cyclic voltammetry (CV), chronopotentiometry parameters, and electrochemical impedance spectroscopy (EIS) were used to study the electrochemical performance of materials.

## Results and discussion

**Figure 1:**
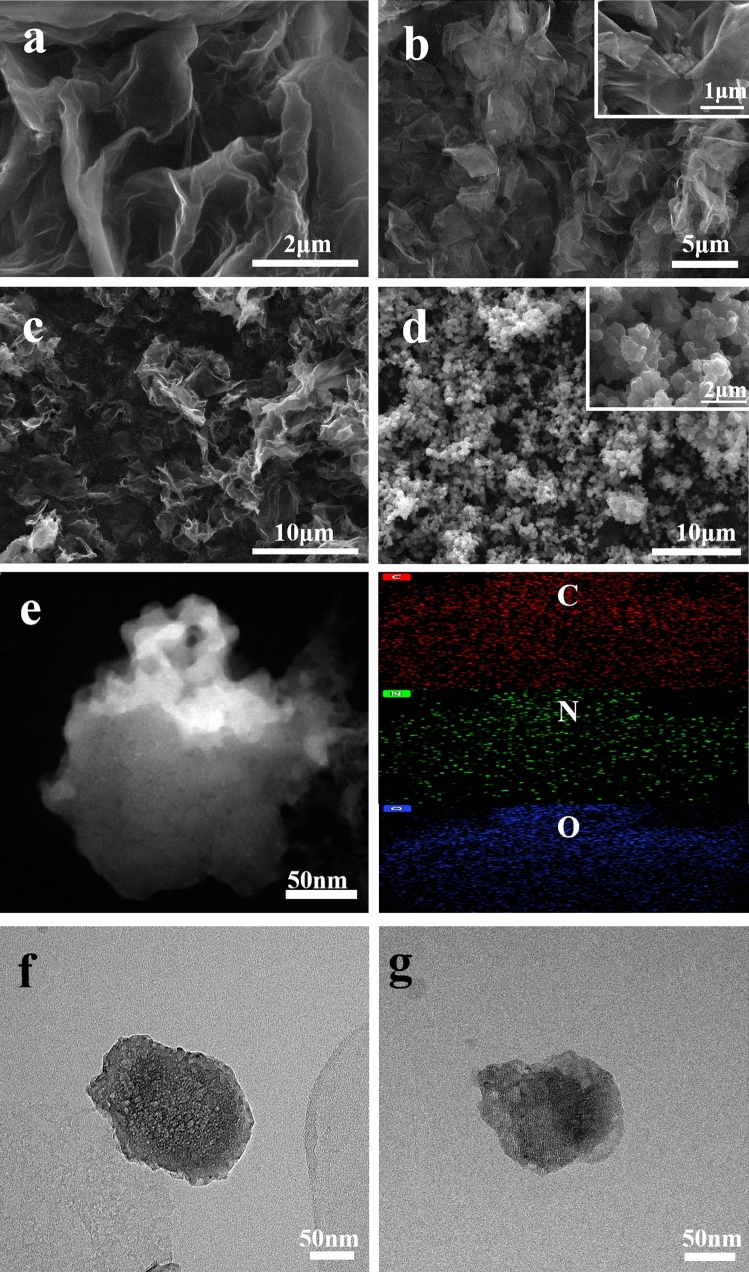
SEM images of GO (**a**), PPy/GO (**b**), N-PCM (**c**), and PPy/N-PCM (**d**), TEM images of N-PCM (**e**, **f**) and PPy/N-PCM (**g**), Elemental mapping images of N-PCM.

Figure 1 presents SEM images of GO, PPy/GO, N-PCM, PPy/N-PCM and TEM images of N-PCM, PPy/N-PCM. As shown in Fig. 1a, the modified Hummers method was successfully used to prepare GO with wrinkles of multiple layers. This not only facilitates the transmission of electrons, but also provides more nucleation sites for PPy^27^. The surface of GO sheet remains smooth before PPy is compounded. PPy nanospheres are deposited on GO nanosheets by in-situ polymerization to form a flower-like structure^9^ (Fig. 1b). The π–π interaction between PPy and GO makes PPy/GO an excellent precursor sample. After PPy/GO is carbonized and activated, N-PCM is obtained. The N-PCM is obtained as shown in Fig. 1c. Figure 1e further shows the microstructure of the N-PCM, the porous structure can effectively adsorb pyrrole particles by electrostatic force because of the increased specific surface area and active sites. The elemental mapping analysis of C, N, and O is performed on N-PCM. The results are shown in the figure. It can be seen that N elements are evenly distributed in N-PCM. It can be observed through SEM that the activated carbon matrix has uniform porous structure, which not only increases its specific surface area, but also promotes rapid migration of electrolyte ions. From Fig. 1d, it is found that the PPy nanospheres are uniformly distributed on the porous carbon matrix, and the porous structure of the carbon matrix is still clearly visible after the polymerization. This is consistent with the results of subsequent electrochemical performance tests. Figure 1f further illustrates the porous structure of the carbon matrix, while Fig. 1g shows that the PPy nanospheres are coated on the surface of the carbon matrix.

**Figure 2:**
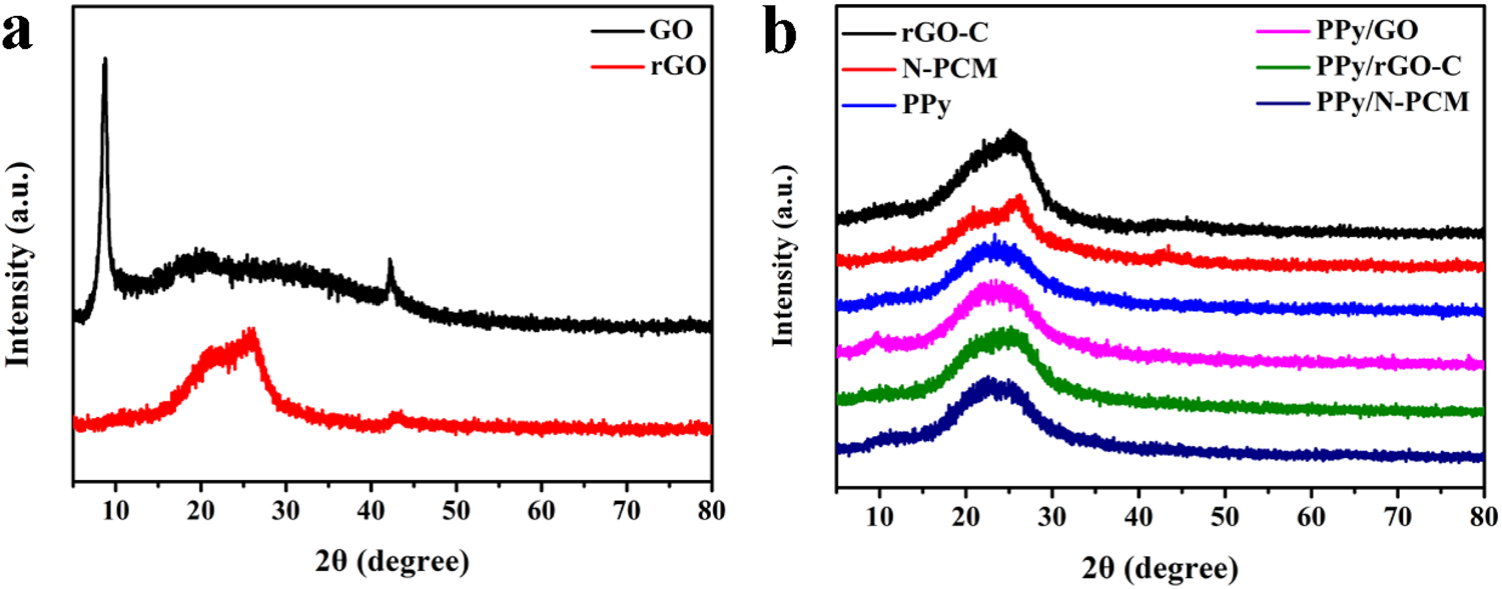
XRD patterns of GO and rGO (**a**) and XRD patterns of rGO-C, N-PCM, PPy, PPy/GO, PPy/rGO-C and PPy/N-PCM (**b**).

Figure 2 presents XRD patterns of GO, rGO, rGO-C, N-PCM, PPy, PPy/GO, PPy/rGO-C and PPy/N-PCM. As shown in Fig. 2a, the diffraction peak at 2θ = 9.78° corresponds to the GO nanosheet (001) crystal plane^28^. This is because a large amount of oxygen-containing functional groups are introduced into the GO nanosheets, which causes the original graphite crystal structure destroyed and increased crystal defects. The diffraction peak at the 9.78° position indicates that the graphite is sufficiently oxidized. In addition, the rGO spectrum shows two diffraction peaks at 2θ = 26.39° and 43.19° that don’t appear in GO, which means that GO has recovered^29^. The peak position of the samples of rGO-C and N-PCM in Fig. 2b are similar to that of rGO in Fig. 2a, which indicates that the carbon matrix after carbonization and doping still maintains the same crystal plane. Compared with rGO-C, the peak intensity of the diffraction peak of N-PCM at 20.78° and 25.97° is weakened. This is because nitrogen atoms are introduced into the graphene lattice, reducing the restacking of graphene sheets^30^. The diffraction peak of GO in the sample of PPy/GO disappeared significantly, demonstrating that the GO surface was completely covered by PPy. The peak positions of all composite samples are exactly the same as that of pure PPy, all of which are located at the characteristic diffraction peaks of pure PPy 2θ = 20°–30°. This indicates that PPy is completely deposited on the surface of the carbon matrix^31^.

**Figure 3:**
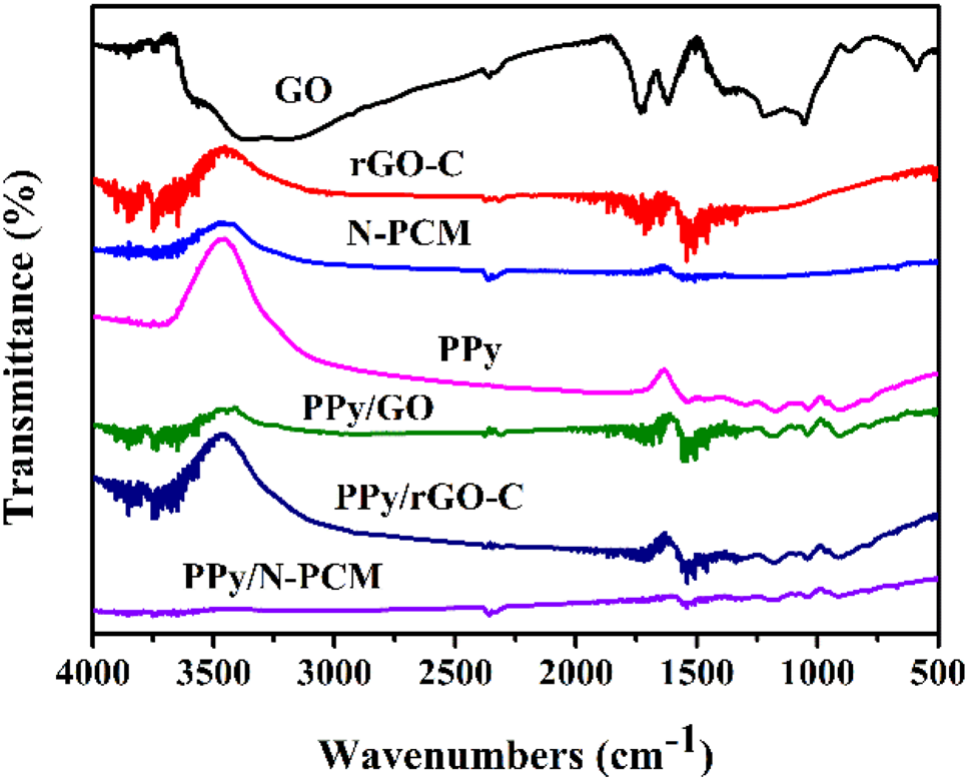
FT-IR spectra of GO, rGO-C, N-PCM, PPy, PPy/GO, PPy/rGO-C and PPy/N-PCM.

FT-IR spectroscopy was performed on all samples to obtain detailed chemical structure information. As shown in Fig. 3, GO shows the absorption peak near 3400 cm^−1^, which is due to the stretching vibration of –OH. In addition, GO exhibits absorption peaks at 1715 cm^−1^ and 1613 cm^−1^, mainly due to the stretching vibrations of C–O and C=C. The diffraction peak of GO near 1371 cm^−1^ is related to the deformation vibration of O–H^17^. The peak of C–O bond in the graphene structure also appeared at 1052 cm^−1^. The spectrum of PPy shows strong absorption peaks at 1537 cm^−1^, 1695 cm^−1^, 1441 cm^−1^, and 1301 cm^−1^, which are attributed to the stretching vibrations of C–C, C=C and C–N in pyrrole ring and C–H deformation vibrations. In addition, peaks at 957 cm^−1^ and 766 cm^−1^ confirm the presence of polymeric pyrrole^32^. The rGO-C and N-PCM have similar characteristic peaks to GO, indicating that they contain similar functional groups. The samples of PPy/GO, PPy/rGO-C and PPy/N-PCM have similar absorption peaks to pure PPy and only have little offset, this further indicating that PPy are successfully anchored on carbon matrix and also maintain the original characteristics of PPy, which is consistent with the XRD analysis results.

**Figure 4:**
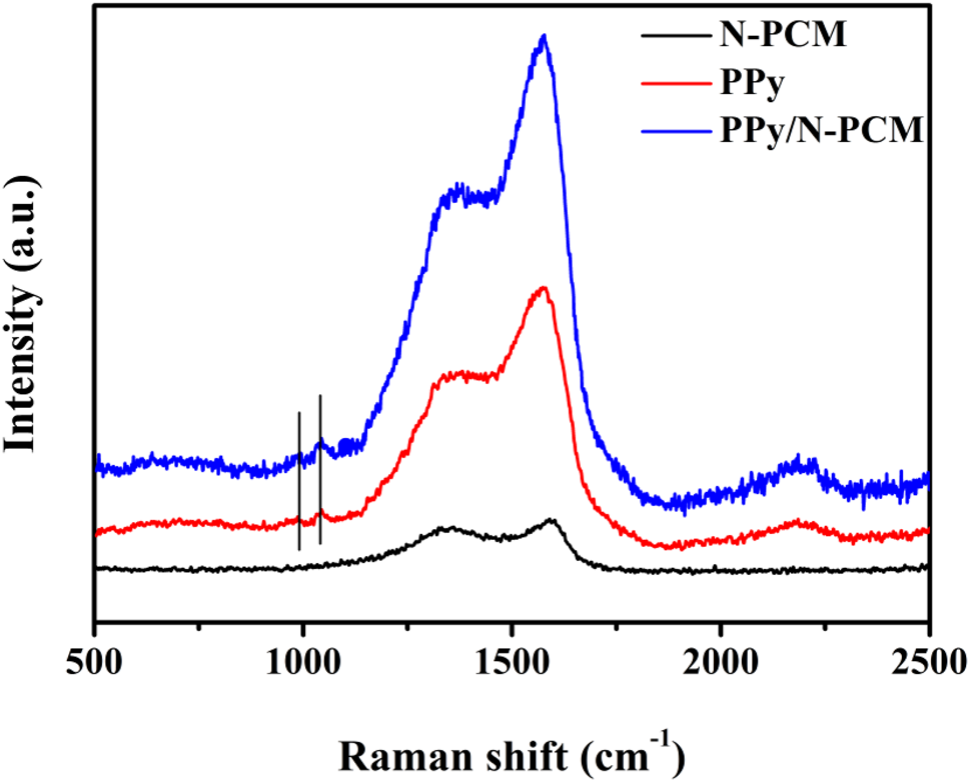
Raman spectra of N-PCM, PPy and PPy/N-PCM.

Figure 4 shows the Raman spectra of N-PCM, PPy and PPy/N-PCM. As shown in the spectrum of N-PCM, the peak of the D band appears at 1350 cm^−1^, and the G band appears at 1580 cm^−1^^33^. The D peak is the structure caused by the defect, and the G peak is attributed to the in-plane stretching vibration of sp^2^ hybridized carbon atoms on the ring and chain. The intensity ratio (I_D_/I_G_) of the D peak to the G peak of the sample N-PCM is 0.908, which indicates that the carbonization and activation treatment can provide the sample with abundant structural defects. The PPy/N-PCM sample shows unique peaks of PPy at 980 cm^−1^ and 1050 cm^−1^^34^. The sample of PPy/N-PCM retains the characteristic peaks of the N-PCM carbon matrix, which confirms that PPy was successfully compounded on the surface of the carbon matrix. The results are consistent with FT-IR.

**Figure 5:**
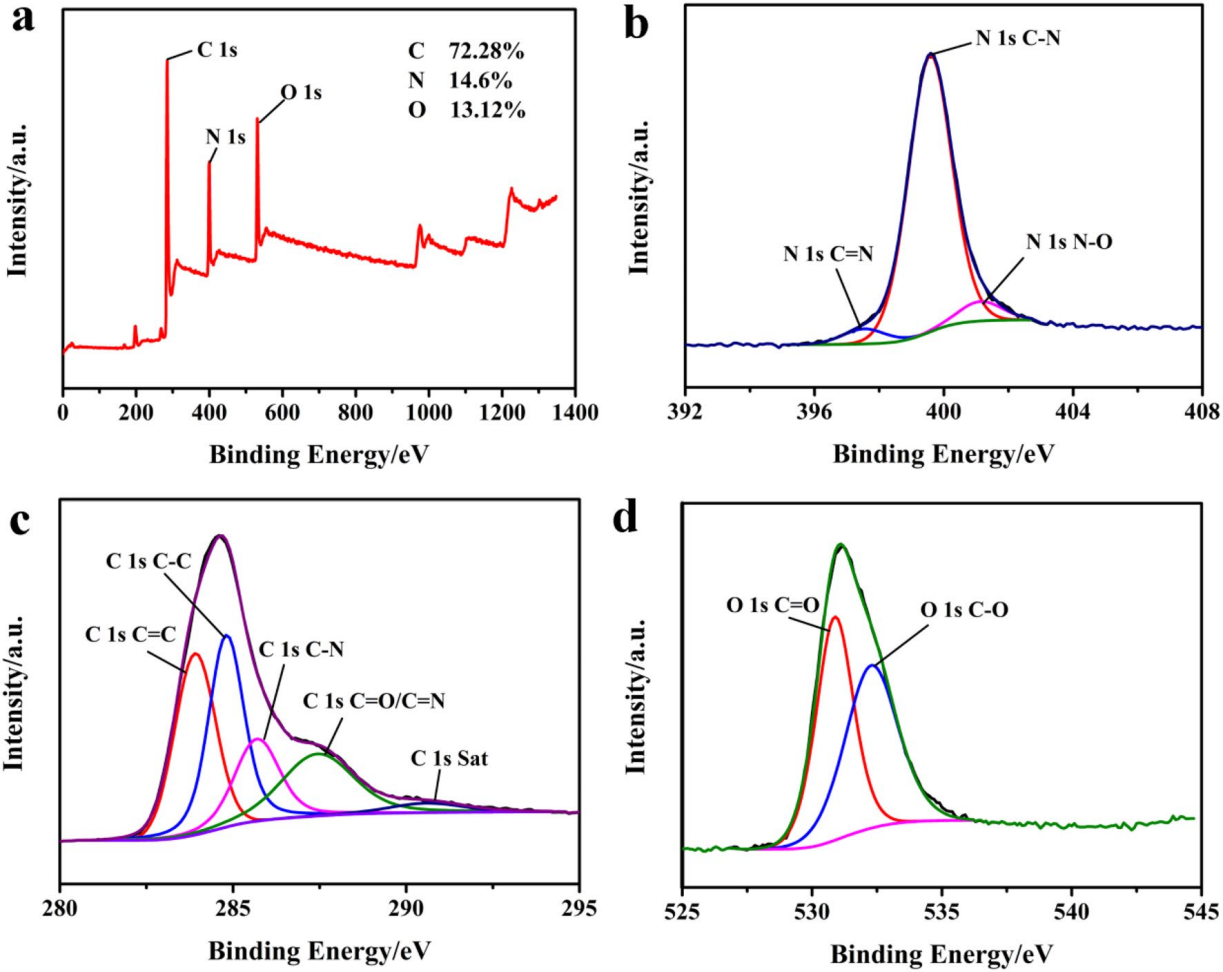
Full XPS spectrum of PPy/N-PCM (**a**); High-resolution XPS spectra of N 1s (**b**), C 1s (**c**) and O 1s (**d**).

Figure 5a shows that the PPy/N-PCM sample mainly contains O 1s, N 1s and C 1s signals. The surface atomic percentages of C, N and O in the sample are 72.28%, 14.6% and 13.12%, respectively. In Fig. 5c, the C 1s shows the C=C chemical state of 283.9 eV, C–C of 284.8 eV, C–N/C–O of 285.69 eV, and the chemical state of C=N/C=O of 287.44 eV coexist. The peak near 290.58 eV is the satellite peak of C 1s, reflecting molecular structure information (It may be the pi-pi* transition of the benzene ring)^35^. This is consistent with the C 1s peak information of N-PCM and PPy in Fig. S1c and Fig. S2c, respectively. The O 1s is shown in Fig. 5d as 530.87 eV C=O and 532.28 eV C–O chemical states coexist. In Fig. 5b, The N is shown as the coexistence of C=N of 397.53 eV, C–N of 399.57 eV and N–O of 401.14 eV. This is consistent with the N 1s peak of PPy in Fig. S2b, indicating that PPy is successfully anchored on the surface of the N-PCM carbon matrix.

**Figure 6:**
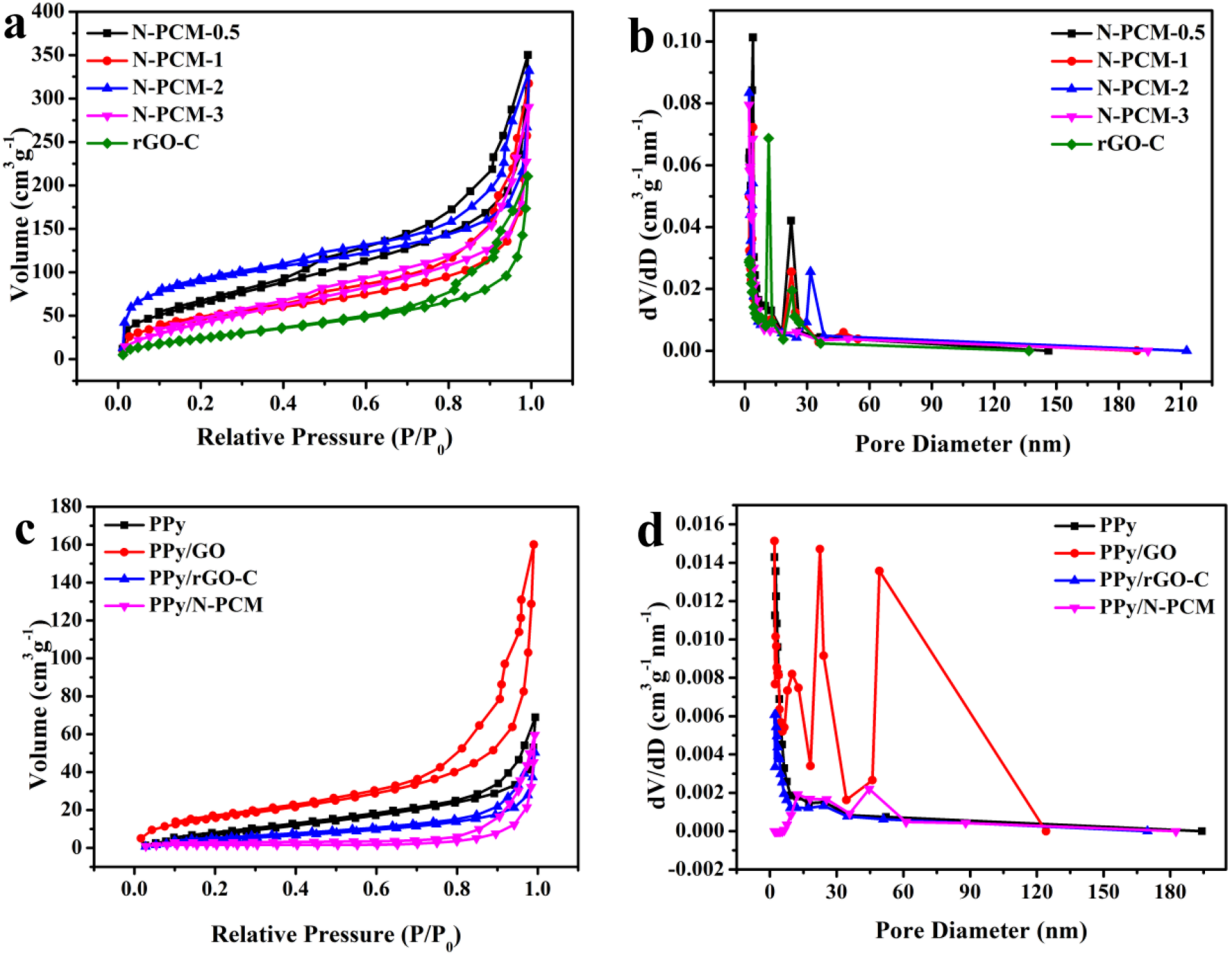
N_2_ adsorption/desorption isotherms of N-PCM-0.5, N-PCM-1, N-PCM-2, N-PCM-3 and rGO-C (**a**) and PPy, PPy/GO, PPy/rGO-C and PPy/N-PCM (**c**); pore size distribution of N-PCM-0.5, N-PCM-1, N-PCM-2, N-PCM-3 and rGO-C (**b**) and PPy, PPy/GO, PPy/rGO-C and PPy/N-PCM (**d**) at 77 K.

In Fig. 6, the adsorption amount of all isotherms increases with the growth of relative pressure. A curve is saw in the latter section and the adsorption hysteresis loop appears in the middle section. This is a typical type IV isotherm characteristic. The pore size distribution curve (Fig. 6b) also indicates the microscopic and pore properties of the samples. The existence of pore structure is related to the release of volatile substances during the carbonization of the polymer^36^. The specific surface area and total pore volume of the corresponding samples are shown in Table 1. When the mass ratio of KOH to C is 2, the obtained carbon matrix has specific surface area of 310 m^2^ g^−1^, and the pore size distribution curve is shown in Fig. 6b. It is apparent that the N-PCM-2 has the highest BET specific surface area and narrow pore size distribution compared to the rest of carbon matrixes. Therefore, N-PCM (N-PCM-2) is a suitable choice as composite precursor. The specific surface area of the composite covered by polypyrrole is reduced compared to carbon skeleton, which mainly due to the deposition of PPy and the filling of pores on surface of carbon substrate by PPy^37,38^.

**Table 1:** Comparison of BET data of different samples.

Sample	rGO-C	N-PCM-0.5	N-PCM-1	N-PCM-2	N-PCM-3	PPy	PPy/GO	PPy/rGO-C	PPy/N-PCM
S_BET_ (m^2^ g^−1^)	99	245	171	310	179	38	60	21	5
Total pore volume (cm^3^ g^−1^)	0.325	0.541	0.491	0.513	0.448	0.106	0.247	0.077	0.092

**Figure 7:**
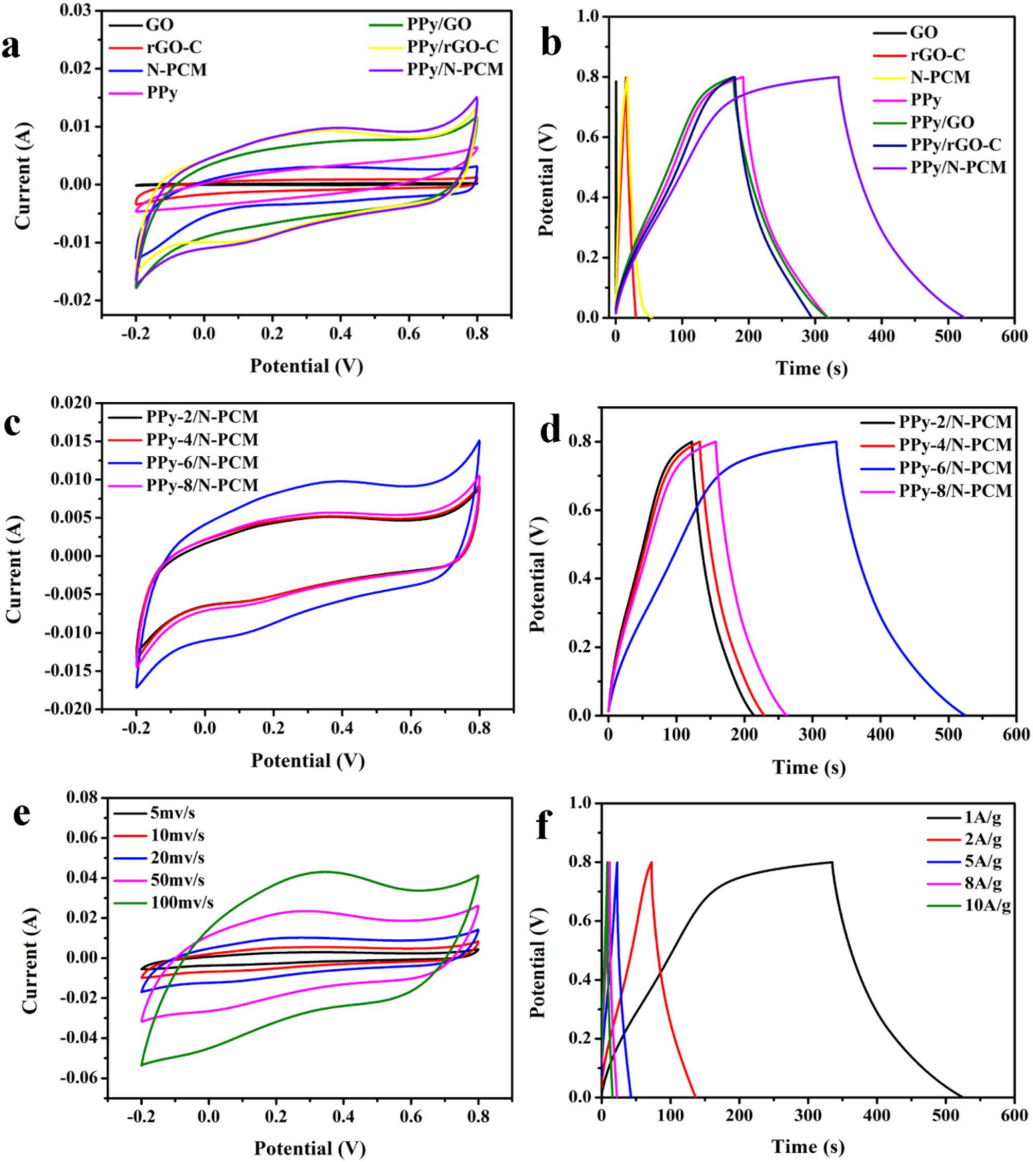
CV curves of GO, rGO-C, N-PCM, PPy, PPy/GO, PPy/rGO-C and PPy/N-PCM at a scan rate of 20 mV s^−1^ (**a**), GCD curves of GO, rGO-C, N-PCM, PPy, PPy/GO, PPy/rGO-C and PPy/N-PCM at the current density of 1 A g^−1^ (**b**); CV curves of PPy/N-PCM with different concentration at a scan rate of 20 mV s^−1^ (**c**); GCD curves of PPy/N-PCM with different concentration at the current density of 1 A g^−1^ (**d**); CV curves of PPy/N-PCM at different scan rates (**e**); and GCD curves of PPy/N-PCM at different current density (**f**).

Figure 7a shows the CV curves of seven different samples at a scan rate of 20 mV s^−1^ and the potential window of − 0.2 to 0.8 V (with Ag/AgCl). As the picture shows, the CV curve of the carbon matrix is like a rectangle while slightly deformed. This is due to the redox reaction on the surface of carbon matrix^39^. The CV curve trend of the samples compounded with PPy is consistent with pure PPy, and both have two redox peaks, indicating that the carbon skeleton and polypyrrole are well combined and have good pseudocapacitance characteristics^40^. Compared with other samples, the CV curve surrounding area of PPy/N-PCM is significantly increased, indicating that the sample of PPy/N-PCM has higher specific capacitance. Figure 7b shows the GCD curves of the seven samples. All of the GCD curves show an approximate regular triangle, indicating that the surface of all of the fabricated electrode materials undergo redox reaction^41^. This is consistent with the CV test results. The specific capacitance value of the discharge part could be calculated according to the following formula^39^:

1 $${\text{C}}_{{\text{m}}} = {\text{ It}}/{\text{m}}\Delta {\text{V}}$$

At current density of 1 A g^−1^, the specific capacitances of GO, rGO-C, N-PCM, PPy, PPy/GO, PPy/rGO-C and PPy/N-PCM are 0.675 F g^−1^, 18.625 F g^−1^, 46.75 F g^−1^, 159.125 F g^−1^, 178.125 F g^−1^, 144.625 F g^−1^ and 237.5 F g^−1^. It can be seen that N-PCM is much higher than the capacitance of GO and rGO-C. This is due to the increase of pore structure on the surface of N-PCM, which increases the effective area required for ion transfer and shortens the ion transfer path. Compared with other literatures, it is found that the specific capacitance of PPy/N-PCM is superior to many other PPy composite materials. (Such as: Oh et al.^42^ prepared SWNT-PPy composite material with a specific capacitance of only 131 F g^−1^. The specific capacitance of the P-CNT/PPy composite prepared by Yang et al. is 188 F g^−1^^43^.) It can be seen from Fig. 7b that the discharge time of PPy/N-PCM is significantly larger than that of other electrode materials, which demonstrates the excellent capacitance characteristics of the PPy/N-PCM, and proves the synergistic effect between PPy and N-PCM.

Figure 7c reveals the effect on electrochemical performance of electrode materials with different pyrrole concentrations. As the Fig. 7c shows, when the concentration of pyrrole is 0.06 M, the area surrounded by CV curve is the largest, and a significant redox peak appears, indicating that PPy-6/N-PCM has the most excellent electrochemical performance. Combined with Fig. 7d, it is found that the 0.06 M PPy/N-PCM discharge time is longer than other samples. Calculated by the formula (1): the specific capacitance of 0.02 M, 0.04 M, 0.06 M and 0.08 M PPy/N-PCM are 114.375 F g^−1^, 118.875 F g^−1^, 237.5 F g^−1^, and 130.5 F g^−1^, respectively. The result is consistent with Fig. 7c. This is because the specific surface area of the porous carbon matrix is increased, so that more polypyrrole nanospheres are embedded on the surface of the carbon matrix. At the same time, the pyrrole monomer concentration of 0.06 M allows the polypyrrole to be uniformly anchored on the surface of the carbon matrix without stacking.

Figure 7e shows the cyclic voltammetry curves for PPy/N-PCM at different scan rates. The area of the CV curve of PPy/N-PCM increases as the scan rate increases. It indicates that the prepared composite sample has good rate performance, and an increase in the scanning rate causes a slight change in shape due to the resistance of the electrode. The symmetrical GCD curves at different current densities indicate that the PPy/N-PCM has good capacitance characteristics (Fig. 7f). When the current density is increased to 10 A g^−1^, the GCD curve of PPy/N-PCM remains unchanged, further confirming that it has high rate performance. Such excellent specific capacitance of the electrode is due to the functionalization of carbon matrix by nitrogen doping. In addition, the presence of nitrogen groups increases the tantalum capacitance response, which in turn contributes to the increased energy storage^44–46^.

**Figure 8:**
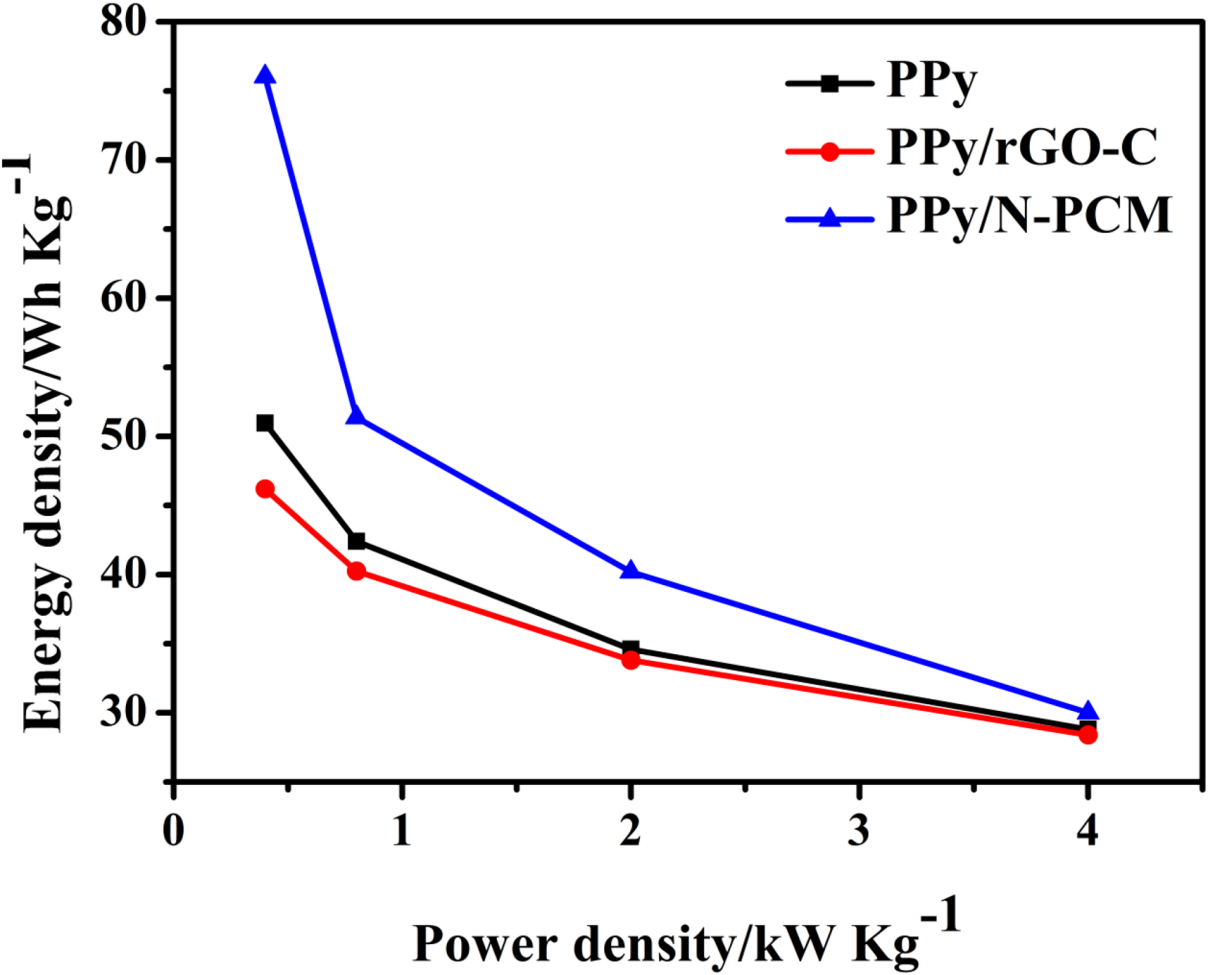
The Ragone plots of PPy, PPy/rGO-C and PPy/N-PCM.

Figure 8 shows the Ragone plots of the samples of PPy, PPy/rGO-C, and PPy/N-PCM. It is worth noting that the PPy/N-PCM has an energy density of 76.04 Wh kg^−1^ at a power density of 0.4 kW kg^−1^, which is greater than PPy and PPy/rGO-C. This shows that the carbon support can effectively control the volume change of PPy, thereby improving its electrochemical performance. Comparing PPy/N-PCM and PPy/rGO-C, it was found that the nitrogen-doped carbon matrix was subjected to alkali-activated pore-forming and then compounded with polypyrrole, the sample had higher energy density. This is because the specific surface area of the carbon matrix after pore formation becomes larger than rGO-C so that PPy can be uniformly compounded on the carbon matrix. In addition, it can be found from the figure that the rate performance of PPy/N-PCM is not as good as that of pure PPy. This may be due to the support of the N-PCM matrix, PPy nanospheres can only grow along the template. This will reduce the specific surface area of the sample to a certain extent, thereby affecting the ion transport and transfer of the PPy/N-PCM sample in the electrolyte. When the high current density is 10 A g^−1^, the power density is 4 kW kg^−1^, and the energy density is 30 Wh kg^−1^, which indicates that the PPy/N-PCM composite has objective application prospects as an electrode material for supercapacitors.

**Figure 9:**
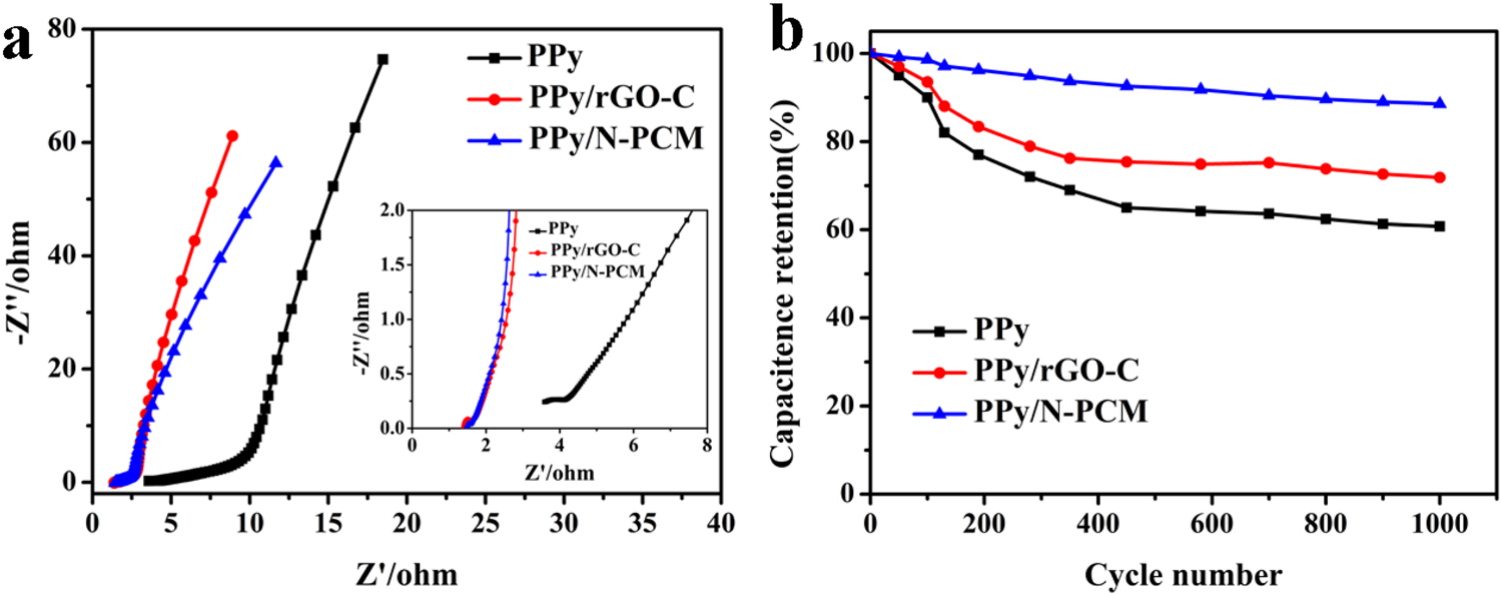
Electrochemical impedance spectroscopy (**a**) and cycle stability at 10 A g^−1^ current density (**b**) of PPy/N-PCM, PPy/rGO-C and pure PPy.

The EIS analysis of the PPy/N-PCM, PPy/rGO-C and PPy were used to study the conductivity and charge transport behavior. The result is shown in Fig. 9a. It is observed that the radius of the high frequency region of PPy/N-PCM and PPy/rGO-C are smaller than the radius of PPy, indicating that the charge transfer resistance is small^47^. The slope of the oblique line presented in the low frequency region is large, indicating that the diffusion rate of ions in electrolyte is very fast^48^. The cycling stability of PPy, PPy/rGO-C and PPy/N-PCM electrodes was tested at a current density of 10 A g^−1^. As can be seen from Fig. 9b, the PPy/N-PCM has excellent cycle stability and still maintains an initial specific capacitance of 88.53% after 1000 cycles of testing. This is attributed to the synergy between PPy nanospheres and N-PCM which inhibits the expansion and contraction of polypyrrole during charge and discharge. The PPy/rGO-C and PPy electrodes only retained 71.84% and 60.76% of the initial capacitance, demonstrating good stability of the PPy/N-PCM. Therefore, PPy/N-PCM composite has better cycle stability than pure PPy nanospheres. The PPy/N-PCM will be an ideal for high-performance energy storage devices.

## Conclusions

In summary, we have continuously tried to develop a new method to synthesize conductive composites with 3D structure. The synthesized PPy/N-PCM was proved to have an excellent three-dimensional structure by SEM, TEM, FT-IR, Raman, XPS and XRD characterization. The surface of the porous carbon matrix provides active sites, which is beneficial to the successful anchoring of PPy and improves utilization of PPy, so that the PPy nanospheres morphology is well controlled. In the electrochemical test, the specific capacitance of PPy/N-PCM reached 237.5 F g^−1^ due to the synergistic effect of PPy and N-PCM. When the current is expanded to 10 A g^−1^ in cycle stability test, the capacitance retention rate can reach 88.53%. The sample of PPy/N-PCM not only solves the morphology problem of polypyrrole, but also increases specific capacitance of carbon matrix. This may be another major breakthrough in the field of conductive polymers.

## Supplementary information

Supplementary information 1

Supplementary information 2

## References

[1] Chen GZ (2016). Supercapacitor and supercapattery as emerging electrochemical energy stores. Int. Mater. Rev..

[2] Zhou H (2016). A highly flexible solid-state supercapacitor based on the carbon nanotube doped graphene oxide/polypyrrole composites with superior electrochemical performances. Org. Electron..

[3] Vangari M (2013). Supercapacitors: review of materials and fabrication methods. J. Energy Eng..

[4] Zhong C (2015). A review of electrolyte materials and compositions for electrochemical supercapacitors. Chem. Soc. Rev..

[5] Chen H (2019). Carbon nanotubes enhance flexible MXene films for high-rate supercapacitors. J. Mater. Sci..

[6] Wang G (2012). A review of electrode materials for electrochemical supercapacitors. Chem. Soc. Rev..

[7] Largeot C (2008). Relation between the ion size and pore size for an electric double-layer capacitor. J. Am. Chem. Soc..

[8] Liu T (1997). Self-discharge and potential recovery phenomena at thermally and electrochemically prepared RuO_2_ supercapacitor electrodes. Elettrochim. Acta.

[9] Huang Y (2016). Nanostructured polypyrrole as a flexible electrode material of supercapacitor. Nano Energy.

[10] Bose S (2011). Carbon-based nanostructured materials and their composites as supercapacitor electrodes. J. Mater. Chem..

[11] Sun J (2016). High-performance stretchable yarn supercapacitor based on PPy@CNTs@urethane elastic fiber core spun yarn. Nano Energy.

[12] Zhu J (2015). Effect of various electrolyte cations on electrochemical performance of polypyrrole/RGO based supercapacitors. Phys. Chem. Chem. Phys..

[13] Cherusseri J (2016). Ultra-flexible fibrous supercapacitors with carbon nanotube/polypyrrole brush-like electrodes. J. Mater. Chem. A.

[14] Lee H (2011). Fabrication of polypyrrole (PPy)/carbon nanotube (CNT) composite electrode on ceramic fabric for supercapacitor applications. Electrochim. Acta.

[15] Fan L-Q (2014). Asymmetric supercapacitor based on graphene oxide/polypyrrole composite and activated carbon electrodes. Electrochim. Acta.

[16] Feng H (2014). Polypyrrole/hexadecylpyridinium chloride-modified graphite oxide composites: fabrication, characterization, and application in supercapacitors. J. Power Sources.

[17] Chang HH (2012). Electrochemically synthesized graphene/polypyrrole composites and their use in supercapacitor. Carbon.

[18] Ezeigwe ER (2015). Solvothermal synthesis of graphene-MnO_2_ nanocomposites and their electrochemical behavior. Ceram. Int..

[19] Zhang D (2013). Preparation of a three-dimensional ordered macroporous carbon nanotube/polypyrrole composite for supercapacitors and diffusion modeling. J. Phys. Chem. C.

[20] Yuan D-S (2011). Nitrogen-enriched carbon nanowires from the direct carbonization of polyaniline nanowires and its electrochemical properties. Electrochem. Commun..

[21] You B (2014). Three-dimensional hierarchically porous all-carbon foams for supercapacitor. ACS Appl. Mater. Interfaces.

[22] Schedin F (2007). Detection of individual gas molecules adsorbed on graphene. Nat. Mater..

[23] Zhang D (2017). Scalable synthesis of hierarchical macropore-rich activated carbon microspheres assembled by carbon nanoparticles for high rate performance supercapacitors. J. Power Sources.

[24] Shi Q (2015). Nitrogen-doped ordered mesoporous carbons based on cyanamide as the dopant for supercapacitor. Carbon.

[25] Chen L (2017). Cotton fabric derived hierarchically porous carbon and nitrogen doping for sustainable capacitor electrode. Carbon.

[26] Hummers WS (1958). Preparation of graphitic oxide. J. Am. Chem. Soc..

[27] Zhang XL (2017). Fabrication of 3D lawn-shaped N-doped porous carbon matrix/polyaniline nanocomposite as the electrode material for supercapacitors. J. Power Sources.

[28] Wang J (2011). Green synthesis of graphene nanosheets/ZnO composites and electrochemical properties. J. Solid State Chem..

[29] Xu J (2010). Hierarchical nanocomposites of polyaniline nanowire arrays on graphene oxide sheets with synergistic effect for energy storage. ACS Nano.

[30] Lin T-T (2015). Graphene-wrapped nitrogen-containing carbon spheres for electrochemical supercapacitor application. J. Anal. Appl. Pyrol..

[31] Peshoria S (2017). Study and explanation about the morphological, electrochemical and structural properties of differently synthesized polypyrrole. J. Mater. Sci. Mater. Electron..

[32] Khoh W-H (2014). Solid-state asymmetric supercapacitor based on manganese dioxide/reduced-graphene oxide and polypyrrole/reduced-graphene oxide in a gel electrolyte. Colloid Surf. A.

[33] Liu A (2010). Electrochemical deposition of polypyrrole/sulfonated graphene composite films. J. Phys. Chem. C.

[34] Bora C (2014). Polypyrrole/sulfonated graphene composite as electrode material for supercapacitor. J. Phys. Chem. C.

[35] Etka M (2019). Raman and XPS studies of ammonia sensitive polypyrrole nanorods and nanoparticles. Sci. Rep..

[36] Hulicova-Jurcakova D (2008). Combined effect of nitrogen-and oxygen-containing functional groups of microporous activated carbon on its electrochemical performance in supercapacitors. Adv. Funct. Mater..

[37] Fan Z (2017). High density of free-standing holey graphene/PPy films for superior volumetric capacitance of supercapacitors. ACS Appl. Mater. Interfaces.

[38] Zhao J (2015). Hydrophilic hierarchical nitrogen-doped carbon nanocages for ultrahigh supercapacitive performance. Adv. Mater..

[39] He H (2019). Fabrication of 3D ordered honeycomb-like nitrogen-doped carbon/PANI composite for high-performance supercapacitors. Appl. Surf. Sci..

[40] Li Y (2013). Oriented arrays of polyaniline nanorods grown on graphite nanosheets for an electrochemical supercapacitor. Langmuir.

[41] Liu C (2016). Synthesis of N-doped hollow-structured mesoporous carbon nanospheres for high-performance supercapacitors. ACS Appl. Mater. Interfaces.

[42] Oh J (2008). Preparation and electrochemical characterization of porous SWNT-PPy nanocomposite sheets for supercapacitor applications. Synth. Met..

[43] Yang L (2015). Polypyrrole directly bonded to air-plasma activated carbon nanotube as electrode materials for high-performance supercapacitor. Electrochim. Acta..

[44] Wang Y (2016). A melamine-assisted chemical blowing synthesis of N-doped activated carbon sheets for supercapacitor application. J. Power Sources.

[45] Lee WH (2014). Monodispersed N-doped carbon nanospheres for supercapacitor application. ACS Appl. Mater. Interfaces.

[46] Tan Y (2013). Synthesis of ultrathin nitrogen-doped graphitic carbon nanocages as advanced electrode materials for supercapacitor. ACS Appl. Mater. Interfaces.

[47] Sk MM (2014). Synthesis of polyaniline nanotubes using the self-assembly behavior of vitamin C: a mechanistic study and application in electrochemical supercapacitors. J. Mater. Chem. A.

[48] Su F (2011). Nitrogen-containing microporous carbon nanospheres with improved capacitive properties. Energy Environ. Sci..

