# Supplementary figures and images for: Synthesis of polypyrrole/nitrogen-doped porous carbon matrix composite as the electrode material for supercapacitors
Source: Sci Rep. 2020 Sep 21;10:15370. doi: 10.1038/s41598-020-72392-x (PMC7505966; doi:10.1038/s41598-020-72392-x)

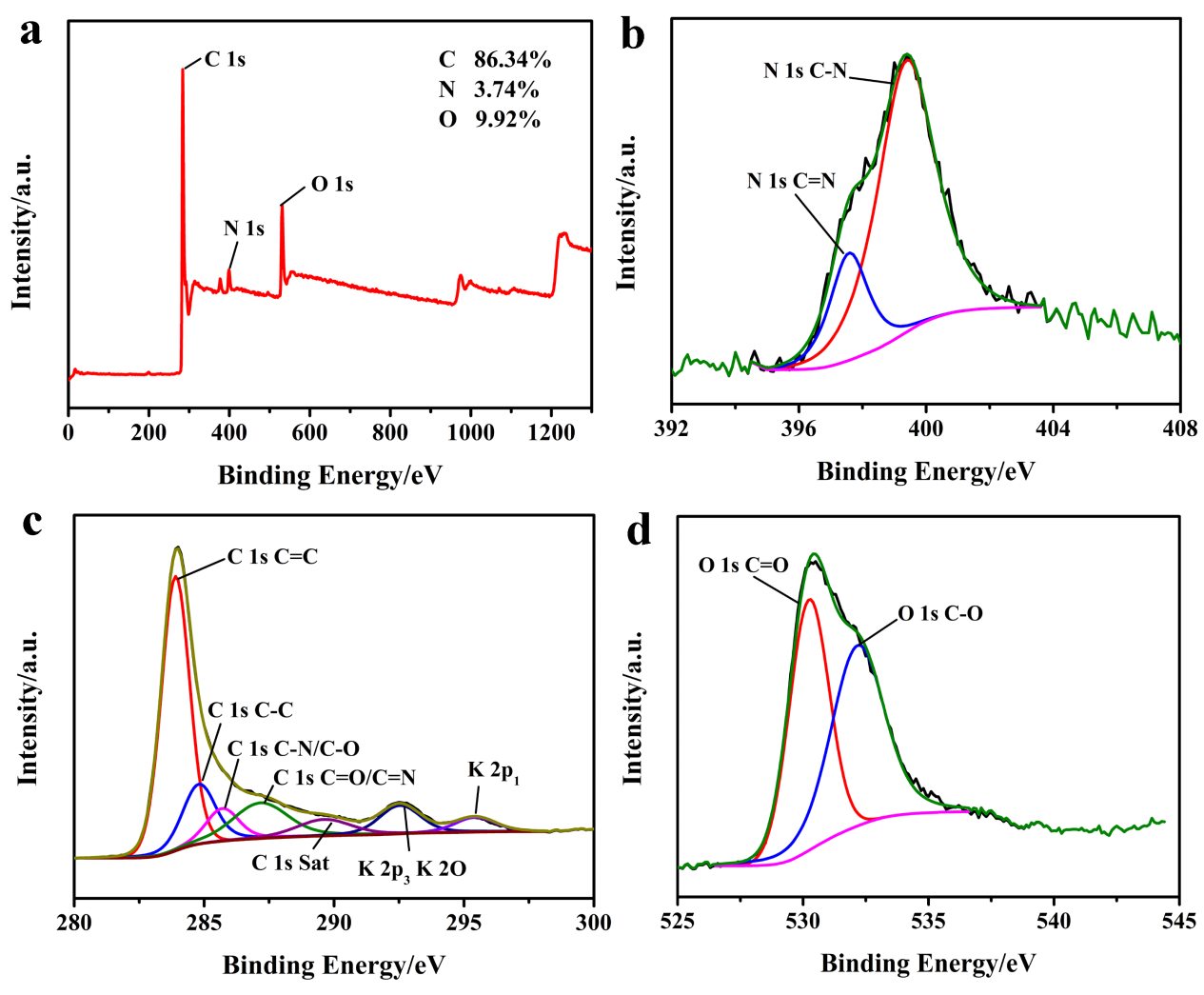


Fig. S1 Full XPS spectrum of N-PCM (a); High-resolution XPS spectra of N 1s (b), C 1s (c) and O 1s (d).

Supplement: Supplementary file 1 — Supplementary information 1 [file 41598_2020_72392_MOESM1_ESM.docx]

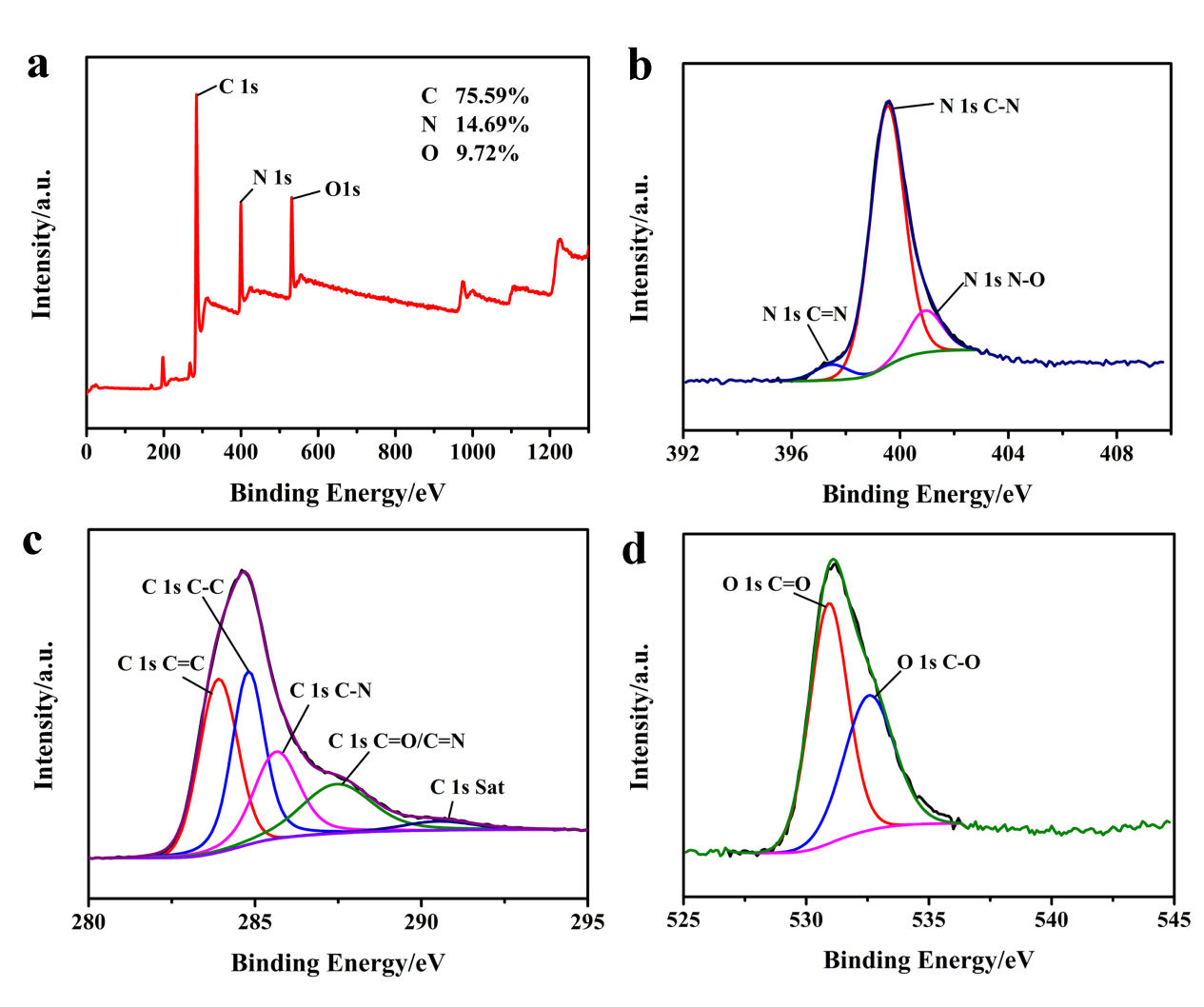


Fig. S2 Full XPS spectrum of PPy (a); High-resolution XPS spectra of N 1s (b), C 1s (c) and O 1s (d).

Supplement: Supplementary file 2 — Supplementary information 2 [file 41598_2020_72392_MOESM2_ESM.docx]
